# 1-*O*-Hexyl-2,3,5-Trimethylhydroquinone Ameliorates l-DOPA-Induced Cytotoxicity in PC12 Cells

**DOI:** 10.3390/molecules24050867

**Published:** 2019-03-01

**Authors:** Hyun Jin Park, Jong Koo Kang, Myung Koo Lee

**Affiliations:** 1 Department of Pharmacy and Research Center for Bioresource and Health, College of Pharmacy, Chungbuk National University, 194-21, Osongsaengmyung 1-ro, Osong, Heungduk-gu, Cheongju 28160, Korea; bwind77@hanmail.net; 2 Department of Veterinary Medicine, College of Veterinary Medicine, Chungbuk National University, 1, Chungdae-ro, Seowon-gu, Cheongju 28644, Korea; jkkang@chungbuk.ac.kr

**Keywords:** 1-*O*-hexyl-2,3,5-trimethylhydroquinone, l-DOPA-induced cytotoxicity, superoxide dismutase, ERK1/2, JNK1/2, PC12 cells

## Abstract

1-*O*-Hexyl-2,3,5-trimethylhydroquinone (HTHQ) has previously been found to have effective anti-oxidant and anti-lipid-peroxidative activity. We aimed to elucidate whether HTHQ can prevent dopaminergic neuronal cell death by investigating the effect on l-DOPA-induced cytotoxicity in PC12 cells. HTHQ protected from both l-DOPA-induced cell death and superoxide dismutase activity reduction. When assessing the effect of HTHQ on oxidative stress-related signaling pathways, HTHQ inhibited l-DOPA-induced phosphorylation of sustained extracellular signal-regulated kinases (ERK1/2), p38 mitogen-activated protein kinase (MAPK), and c-Jun N-terminal kinase (JNK1/2). HTHQ also normalized l-DOPA-reduced Bcl-2-associated death protein (Bad) phosphorylation and Bcl-2-associated X protein (Bax) expression, promoting cell survival. Taken together, HTHQ exhibits protective effects against l-DOPA-induced cell death through modulation of the ERK1/2-p38MAPK-JNK1/2-Bad-Bax signaling pathway in PC12 cells. These results suggest that HTHQ may show ameliorative effects against oxidative stress-induced dopaminergic neuronal cell death, although further studies in animal models of Parkinson’s disease are required to confirm this.

## 1. Introduction

1-*O*-Hexyl-2,3,5-trimethylhydroquinone (HTHQ), a hydroquinone monoalkyl ether, is a potent antioxidative agent that has been found to exert anti-lipid-peroxidative activity in rat liver microsomes [1] and chemopreventive effects against heterocyclic amine-induced carcinogenesis [2]. HTHQ has considerable anti-oxidative activity by directly reacting with reactive oxygen species (ROS), including peroxyl radicals, and scavenging them to form more stable free radicals [1].

3,4-l-Dihydroxyphenylalanine (l-DOPA) is a highly effective and widely used treatment for Parkinson’s disease (PD) [3]. However, high levels of l-DOPA have been shown to lead to the production of ROS in PC12 and dopaminergic neuronal cells, resulting in oxidative stress-induced cell death [4,5]. Oxidative stress-mediated cell death/apoptosis has been shown to be caused by caspase-cascade activation following phosphorylation of p38 mitogen-activated protein kinase (MAPK), c-Jun N-terminal kinase (JNK1/2), and sustained extracellular signal-regulated kinase activation (ERK1/2) [6,7,8,9]. Modulation of Bcl signaling mediated by Bcl-2-associated death protein (Bad) and Bcl-2-associated X protein (Bax) was also shown to promote cellular survival following oxidative stress [10,11]. 

Both short-term and repeated l-DOPA treatments have been reported to increase intracellular levels of cyclic AMP in PC12 cells, which induced either transient or sustained ERK1/2 phosphorylation [9,12,13]. These studies reported that either a single treatment with l-DOPA at toxic levels or repeated treatments with l-DOPA at non-toxic levels can induce sustained ERK1/2 phosphorylation in PC12 cells which result in apoptotic cell death, while transient ERK1/2 phosphorylation promotes cell survival [9,12]. Therefore, in addition to l-DOPA-induced oxidative stress, ERK activation may be another factor regulating cell survival and death following l-DOPA treatment. 

PC12 cells have dopamine synthesizing, storing and releasing properties similar to those of neurons [14]. Neuronal differentiation of PC12 can also be induced by formation of sympathetic neuron-like neurite outgrowth in the presence of the trophic factors, including nerve growth factor (NGF, 1–20 ng/mL, for 12–24 h) [15]. This capacity to undergo neuronal differentiation in response to NGF is a useful and important feature of PC12 cells for PD studies [4,15]. Therefore, PC12 cells represent an effective in vitro model for the study of dopamine biosynthesis, oxidative-induced cytotoxicity, and NGF-mediated cell differentiation [9,12,13,15], which can be applied for examining the anti-neurodegenerative agents for PD.

As an antioxidant, HTHQ could represent a potential therapeutic agent for oxidative stress-induced cytotoxicity. However, the effects of HTHQ on l-DOPA-induced neurotoxicity, including the underlying signaling mechanisms, have not yet been elucidated. We, therefore, aimed to investigate the effects of HTHQ on l-DOPA-induced cytotoxicity in PC12 cells as a model system in order to elucidate whether it can prevent dopaminergic neuronal cell death.

## 2. Results

### 2.1. Cell Viability

**Figure 1:**
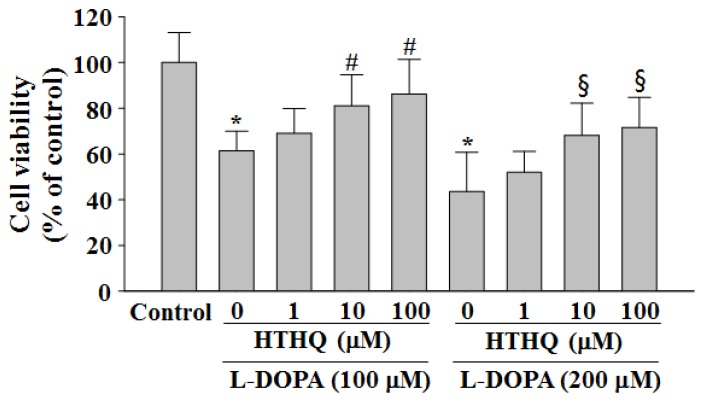
Effects of 1-*O*-hexyl-2,3,5-trimethylhydroquinone (HTHQ) on 3,4-l-dihydroxyphenylalanine (l-DOPA)-induced cell viability in PC12 cells. PC12 cells were exposed to l-DOPA (100 and 200 μM) in the absence or presence of HTHQ (1, 10, and 100 μM) for 24 h. The cell viabilities were assessed using the 3-(4,5-dimethyl-2-thiazolyl)-2,5-diphenyl-2H-tetrazolium bromide (MTT) assay, in which visible cells convert the soluble dye MTT to insoluble blue formazan crystals. The results represent the mean ± S.E.M. (n = 4–6). * *p* < 0.05 compared to the baseline, ^#^ *p* < 0.05 compared to 100 µM l-DOPA-treated group, ^§^ *p* < 0.05 compared to 200 µM l-DOPA-treated group (analysis of variance (ANOVA) with post-hoc Tukey’s test).

Treatment with l-DOPA (100 and 200 μM) reduced cell viability in PC12 cells as previously described to 61.4% and 43.5% of control levels for 24 h, respectively (Figure 1) [9]. However, the reduced cell viability at 24 h caused by both 100 and 200 µM l-DOPA was significantly attenuated by HTHQ treatment at 1, 10, and 100 µM to 69.1%, 81.1% (*p* < 0.05), and 86.2% (*p* < 0.05) (100 µM l-DOPA; 1, 10, and 100 µM HTHQ, respectively), or 51.9%, 68.1% (*p* < 0.05), and 71.6% (*p* < 0.05) (200 µM l-DOPA; 1, 10, and 100 µM HTHQ, respectively) (Figure 1). The cytotoxic intensities of l-DOPA and cell viabilities by HTHQ were concentration-dependent, indicating that the interaction with HTHQ to l-DOPA did not occur. In the cell-free condition, the addition of HTHQ to MTT solution did not cause the changes in the values of optical density, suggesting that HTHQ did not interact directly with MTT. In addition, HTHQ at concentrations up to 500 μM did not show cytotoxicity for 24 h in PC12 cells.

### 2.2. SOD Activity

**Figure 2:**
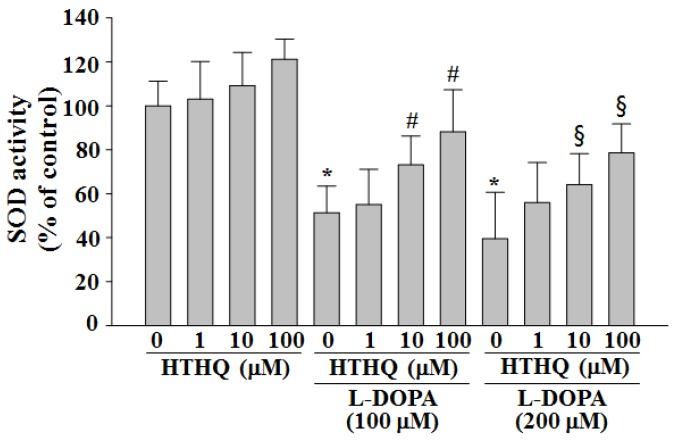
Effect of HTHQ on l-DOPA-induced SOD activity in PC12 cells. PC12 cells were exposed to l-DOPA (100 and 200 μM) in the absence or presence of HTHQ (1, 10, and 100 μM) for 24 h. superoxide dismutase (SOD) activity was measured by SOD assay kit-WST. Results are expressed as the mean ± S.E.M. (n = 4). * *p* < 0.05 compared to the control level, ^#^ *p* < 0.05 compared to 100 µM L-DOPA-treated group, ^§^ *p* < 0.05 compared to 200 µM l-DOPA-treated group (ANOVA with post-hoc Tukey’s test).

HTHQ at concentrations of 1, 10, and 100 μM without treatment with l-DOPA for 24 h did not significantly induce superoxide dismutase (SOD) activity (Figure 2). SOD activity was markedly reduced by 24 h treatment with 100 or 200 µM l-DOPA (decrease to 51.4% and 39.5% of the control level; both *p* < 0.05) (Figure 2), but this decrease was significantly inhibited by co-treatment with HTHQ. HTHQ rescued SOD levels to 55.1%, 73.1% (*p* < 0.05), and 88.2% (*p* < 0.05) of the control level (100 µM l-DOPA; 1, 10, and 100 µM HTHQ, respectively), or 56.0%, 64.1% (*p* < 0.05), and 78.6% (*p* < 0.05) of the control level (200 µM l-DOPA; 1, 10, and 100 µM HTHQ, respectively) (Figure 2).

Next, the effects of HTHQ (10 μM) on the oxidative stress-related signaling pathway using l-DOPA (200 μM) were investigated. 

### 2.3. Phosphorylation of ERK1/2

**Figure 3:**
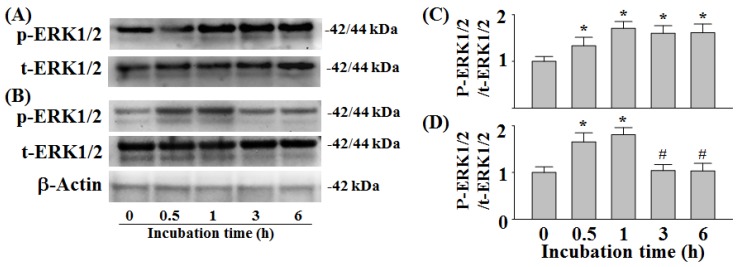
Effect of HTHQ on l-DOPA-induced ERK1/2 phosphorylation in PC12 cells. PC12 cells were exposed to 200 µM l-DOPA (**A**,**C**) in the presence of 10 µM HTHQ (**B**,**D**) for 0.5–6 h. The values of the relative density ratios of ERK1/2 phosphorylation (p-ERK1/2) to total ERK1/2 (t-ERK1/2) are expressed in arbitrary units. The results are expressed as the mean ± S.E.M. (n = 4). * *p* < 0.05 compared to control level (0 h), ^#^ *p* < 0.05 compared to values at 0.5 and 1 h (ANOVA with post-hoc Tukey’s test).

Treatment with 200 µM l-DOPA for 0.5–6 h in PC12 cells has been shown to induce a sustained 1.33–1.71-fold increase in ERK1/2 phosphorylation (Figure 3A,C) [9,13]. We found that following co-treatment of 10 µM HTHQ with 200 µM l-DOPA for 0.5–1 h, ERK1/2 phosphorylation was increased 1.65–1.81-fold compared to the control (sustained ERK1/2 phosphorylation) (Figure 3B,D). However, the ERK1/2 phosphorylation was reduced to baseline levels following 3–6 h of co-treatment (*p* < 0.05 compared to 0.5–1 h) (Figure 3B,D). 

### 2.4. Phosphorylation of p38MAPK and JNK1/2

**Figure 4:**
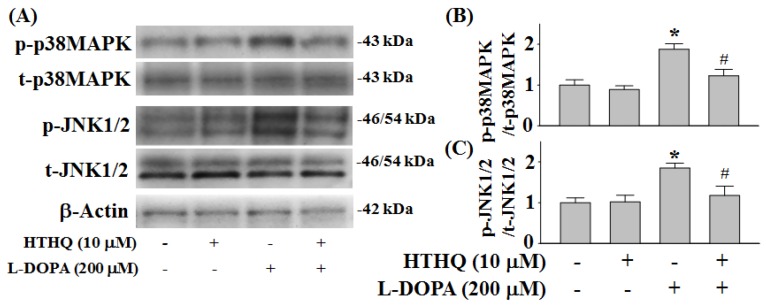
Effect of HTHQ on l-DOPA-induced p38MAPK (**A**,**B**) and JNK1/2 (**A**,**C**) phosphorylation in PC12 cells. PC12 cells were exposed to 200 µM l-DOPA (+) in the absence (−) or presence (+) of 10 µM HTHQ for 6 h. The values of the relative density ratios of p38MAPK phosphorylation (p-p38MAPK) to total p38MAPK (t-p38MAPK) and JNK1/2 phosphorylation (p-JNK1/2) to total JNK1/2 (t-JNK1/2) are expressed in arbitrary units. The results are expressed as the mean ± S.E.M. (n = 4). * *p* < 0.05 compared to l-DOPA-untreated group, ^#^ *p* < 0.05 compared to l-DOPA-treated group (200 μM) (ANOVA with post-hoc Tukey’s test).

Consistent with previous findings, increased p38MAPK and JNK1/2 phosphorylation following 6 h of 200 µM l-DOPA treatment was detected compared to control levels in PC12 cells (Figure 4) [9]. However, co-treatment with 10 µM HTHQ significantly reduced this l-DOPA-induced p38MAPK phosphorylation (*p* < 0.05; Figure 4A,B). Additionally, while HTHQ treatment alone did not affect JNK1/2 phosphorylation, it could significantly reduce l-DOPA-induced JNK1/2 phosphorylation (*p* < 0.05; Figure 4A,C). 

### 2.5. Phosphorylation of Bad and Expression of Bax

**Figure 5:**
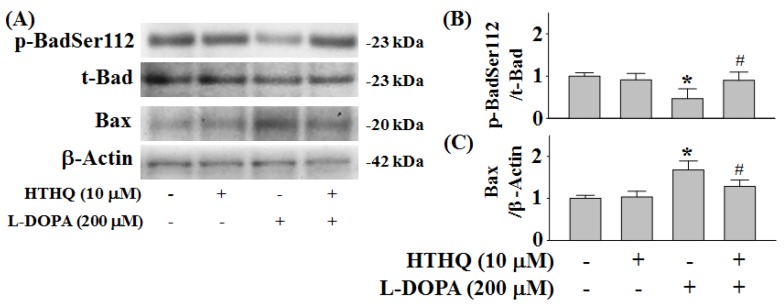
Effect of HTHQ on l-DOPA-induced changes in Bad phosphorylation (**A**,**B**) and Bax (**A**,**C**) expression in PC12 cells. PC12 cells were exposed to 200 µM l-DOPA (+) in the absence (–) or presence (+) of 10 µM HTHQ for 2 h. The values of the relative density ratios of Bad phosphorylation at Ser112 (p-BadSer112) to total Bad (t-Bad) and Bax expression to β-actin are expressed in arbitrary units. The results are expressed as the mean ± S.E.M. (n = 4). * *p* < 0.05 compared to L-DOPA-untreated group, ^#^ *p* < 0.05 compared to L-DOPA-treated group (200 μM) (ANOVA with post-hoc Tukey’s test).

l-DOPA (200 µM) for 2 h reduced Bad phosphorylation at Ser112 (p-BadSer112) by 0.45-fold compared to control levels (*p* < 0.05) (Figure 5A,B), which could be recovered to 0.89-fold by co-treatment with 10 µM HTHQ (*p* < 0.05) (Figure 5A,B). While HTHQ did not induce the expression of Bax at 2 h (Figure 5A,C), it could decrease l-DOPA-induced expression of Bax from 1.68-fold to 1.28-fold of control levels (*p* < 0.05) (Figure 5A,C). 

### 2.6. Expression of Cleaved Caspase-3

**Figure 6:**
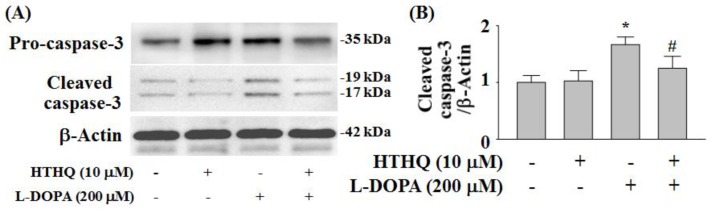
Effects of HTHQ on l-DOPA-induced cleaved caspase-3 expression in PC12 cells. PC12 cells were exposed to l-DOPA (200 µM) (+) in the absence (−) or presence (+) of 10 µM HTHQ for 24 h. The cleaved caspase-3 was analyzed with Western blotting (**A**) and the values of the relative density ratios of cleaved caspase-3/β-actin are expressed in arbitrary units (**B**). The results are expressed as the means ± S.E.M. (n = 4). * *p* < 0.05 compared to l-DOPA-untreated group, ^#^ *p* < 0.05 compared to l-DOPA-treated group (200 µM) (ANOVA with post-hoc Tukey’s test).

HTHQ at 10 µM did not alter the expression of cleaved caspase-3 for 24 h. The expression of cleaved caspase-3 was markedly increased by treatment with l-DOPA (200 µM) for 24 h to 1.67-fold of the control level (*p* < 0.05) (Figure 6A,B) and this effect was significantly reduced by HTHQ (10 µM) to 1.24-fold of the control level for 200 µM l-DOPA (*p* < 0.05) (Figure 6A,B).

## 3. Discussion

HTHQ has been found to act as a potential antioxidative agent by elevating the rat liver microsomal function [1]. In this study, HTHQ was able to ameliorate l-DOPA-induced cytotoxicity, probably by inhibiting the initiation of ROS formation in PC12 cells (Figure 1 and Figure 2), and was not cytotoxic at levels up to 500 µM (data not shown). We next aimed to investigate the modulation of signaling pathways by HTHQ during l-DOPA-induced cytotoxicity. l-DOPA at 100 and 200 μM reduced cell viability to 61.4–43.5% for 24 h in PC12 cells, which was more toxic compared with the previous study [9]. l-DOPA at 100 and 200 μM also induces ERK1/2 activity to ca. 1.6–1.8-fold in PC12 cells [9]. In this study, the same concentrations of l-DOPA induced ERK1/2 phosphorylation to 1.6–1.7-fold. These differences might be caused by the experimental conditions including cell numbers and treatment duration.

ERK1/2, which is known as p42/p44MAPK, is involved in mitogenic signaling, while p38MAPK and JNK are involved in oxidative stress signaling [16]. Short/transient activations of ERK1/2 by epidermal growth factor increase cell proliferation in PC12 cells. Conversely, prolonged or sustained ERK1/2 activation and the nuclear translocation of ERK1/2 induced by nerve growth factor result in the arrest of cellular growth and neuronal differentiation [15], and can induce neurotoxicity [17,18]. In addition, toxic levels of l-DOPA (100 and 200 μM) were previously shown to induce transient ERK1/2 phosphorylation in PC12 cells after treatment for 0.5–1 h, but caused sustained ERK1/2 phosphorylation via cyclic AMP-Epac system after 3–6 h, which resulted in neurotoxicity [13]. High concentrations of l-DOPA (100 and 200 μM) were furthermore shown to elicit cytotoxicity via activation of p38MAPK and JNK1/2 in PC12 cells [9].

l-DOPA also induces activation of ERK1/2 in the dopamine-depleted striatum [19] and in the striatonigral medium spiny neurons of hemiparkinsonian mice, which results in dyskinesia [20]. JNK1/2 correlates with apoptotic neuronal degeneration in PC12 and neuronal cells, which is associated with activation of AP-1 and overexpression of c-Jun or apoptosis signal-regulating kinase-1 [7]. While low doses of l-DOPA do not cause cytotoxicity in PC12 cells [9] and are beneficial for the treatment of PD by increasing dopamine levels in the brain [21], repeated treatments with l-DOPA at non-toxic levels can cause sustained ERK1/2 and JNK1/2 activation in PC12 cells, which in turn induce c-Jun phosphorylation at Ser63 and c-Jun expression and result in cell death [22]. This finding has been confirmed by showing that long-term treatment with low doses of l-DOPA was toxic to dopaminergic neurons in a rat model of PD [22]. Therefore, ERK1/2 activity-modulating agents could be applied to protect from l-DOPA-induced neurotoxicity. In our study, treatment with HTHQ was able to reduce both sustained phosphorylation of ERK1/2 and JNK1/2 phosphorylation to baseline levels following l-DOPA-treatment (Figure 3 and Figure 4A,C).

Furthermore, the regulation of Bax/Bad plays a role in neuronal survival and death. The activity of Bax, a pro-apoptotic factor, can stimulate the release of cytochrome C in neuronal apoptosis [23]. Toxic levels of L-DOPA induce Bad phosphorylation at Ser155, and reduce p-BadSer112 in PC12 cells [9], while non-toxic levels of l-DOPA induce p-BadSer112 [9]. 6-Hydroxydopamine (6-OHDA) is a neurotoxic agent that is induced by oxidative stress and typically formed during long-term therapy with l-DOPA [24]. 6-OHDA has been also shown to reduce p-BadSer112 activation and enhance the Bax expression in PC12 cells [25]. In addition, high/toxic levels of L-DOPA induce apoptotic cell death by increasing cleaved-caspase-3 expression at 24 h in PC12 cells [9]. In this study, toxic levels of l-DOPA activate the Bax expression in PC12 cells (Figure 5A,C). However, the reduced p-BadSer112 and induced Bax expression caused by l-DOPA were normalized by HTHQ treatment. The increase in cleaved-caspase-3 expression was also reduced by HTHQ treatment.

Taken together, these results indicate that HTHQ reverses the pro-apoptotic effects of l-DOPA on multiple signaling pathways that modulate anti-oxidative-related cell survival programs.

Oxidative stress-induced neuronal cell death is a prominent pathogenic component in PD [26]. l-DOPA treatment induces the formation of hydroxyl radicals in dopaminergic neurons in vivo [27], and the production of nitric oxide in the striatum, which has been associated with PD [28]. To improve l-DOPA therapy, it has been suggested to co-administer scavenging agents capable of depleting ROS to protect dopaminergic neurons from l-DOPA-induced cytotoxicity [4,26]. 

SOD activity is increased in parkinsonian substantia nigra in order to eliminate the superoxide anion burst [29]. Patients with PD have significantly higher hydroxyl radical levels and plasma SOD activity, and significantly lower SOD/SOD1 and SOD1 values in red blood cells, which may involve the onset and progression of PD [30]. In addition, SOD protects cells from l-DOPA-induced oxidative cytotoxicity in SH-SY5Y cells [31]. SOD activity is also reduced by treatments with high/toxic levels of l-DOPA and 6-OHDA in PC12 cells [24,32]. It has been reported that, among the ROS, such as superoxide anion radicals, hydroxyl radicals, *t*-butyl peroxyl radicals and singlet oxygens, HTHQ scavenges *t*-butyl peroxyl radicals most effectively by reacting directly with peroxyl radicals [1]. Subsequently, HTHQ exhibits anti-lipid-peroxidative activity by scavenging in peroxides of linolate micelles, liposomes and rat liver microsomes [1].

In this study, HTHQ inhibited l-DOPA-induced decrease in SOD activity in PC12 cells, which can support that HTHQ inhibits the initiation of ROS formation [1]. Anti-oxidative agents such as selegiline, rasagiline, and coenzyme Q10 have previously been applied in vivo therapies [26]. Therefore, based on our data, HTHQ could represent a promising adjuvant therapeutic agent against l-DOPA-induced neurotoxicity by both inhibiting the initiation of ROS formation and modulating the activity of ERK1/2.

## 4. Experimental

### 4.1. Materials

HTHQ were obtained from Biotoxtech Co. (Cheongju, Korea). l-DOPA and 3-(4,5-dimethyl-2-thiazolyl)-2,5-diphenyl-2H-tetrazolium bromide (MTT) were purchased from Sigma–Aldrich (St. Louis, MO, USA). Primary antibodies against ERK1/2, phospho-ERK1/2 (Thr202/Tyr204), p38MAPK at Thr180/Tyr182 [phospho-p38MAPK (Thr180/Tyr182)], JNK1/2, phospho-JNK1/2 (Thr183/Thr185), Bad at Ser112 [phospho-Bad (Ser112)], Bax and β-actin were purchased from Cell Signaling Technology Inc. (Beverly, MA, USA). All sera, antibiotics and RPMI 1640 used for cell culture were obtained from Gibco BRL (Grand Island, NY, USA). All other chemicals were of reagent grade. 

### 4.2. Cell Culture

PC12 cells were grown in RPMI medium 1640 supplemented with 10% heat-inactivated horse serum, 5% heat-inactivated fetal bovine serum, 100 units/mL penicillin, and 100 μg/mL streptomycin. Cells were incubated in a humidified atmosphere with 5% CO_2_ and 95% air at 37 °C as previously described [14].

### 4.3. Measurement of Cell Viability

Cell viability was determined using a conventional MTT assay. PC12 cells were distributed into 96-well plates and incubated for 2 days. Following treatment with l-DOPA in the absence or presence of HTHQ for 24 h, MTT solution was added to each well and incubated at 37 °C for 2 h. Finally, the reaction was stopped by the addition of 0.8 M HCl in isopropanol [13]. The absorbance was then measured at 570 nm using a Bauty Diagnostic Microplate Reader (Molecular Devices, Sunnyvale, CA, USA).

### 4.4. Assay for Superoxide Dismutase (SOD) Activity

Following treatment with l-DOPA in the absence or presence of HTHQ for 24 h, PC12 cells were harvested and lysed in a hypotonic buffer (1% NP-40, 50 mM Tris-HCl, pH 7.5, and 0.05 mM EDTA) for 20 min at 4 °C. The lysates were centrifuged at 15,000× *g* for 10 min, and SOD activity was determined in an aliquot of the supernatant by using the SOD Assay Kit-WST (Dojindo, Rockville, MD, USA) as described previously [33]. SOD activity was calculated according to the manufacturer’s instructions on the basis of a difference in absorbance between the standard and each sample. The SOD activity was expressed as a percentage after being adjusted by the amount of units/mg protein in each sample [25].

### 4.5. Western Blot Analysis

Following treatment with l-DOPA in the absence or presence of HTHQ for 0.6–24 h, PC12 cells (ca. 1 × 10^6^ cells/mL) were collected and homogenized at 4 °C for western blot analysis. Phosphorylation of ERK1/2 at Thr202/Tyr204 (p-ERK1/2, Thr202/Tyr204), JNK1/2 at Thr183/Thr185 (p-JNK1/2, Thr183/Thr185), and Bad at Ser112 (p-BadSer112) and total Bad (t-Bad) and β-actin, and Bax, caspase-3 and cleaved-caspase-3 expression were determined using western blot analysis as previously described [12,13]. Protein samples (20 μg in each lane) were separated by electrophoresis, incubated with primary antibodies (1:1000 in Tris-buffered saline (TBS-T) with 5% bovine serum albumin (BSA)) at 4 °C, and subsequently incubated with secondary antibodies (1:5000 in TBS-T with 5% BSA). Bands were visualized using the enhanced chemiluminescence kit (Amersham Pharmacia Biotech, Piscataway, NJ, USA) and captured on a radiographic film [13].

### 4.6. Statistical Analysis

All data are presented as the mean ± S.E.M. of at least four independent experiments. Protein amounts were determined with a bicinchoninic acid protein assay kit using BCA (Pierce Protein Research Products, Rockford, IL, USA). Statistical analysis was performed using one-way ANOVA followed by post-hoc Tukey’s test, and a *p*-value < 0.05 was considered to indicate statistical significance.

## 5. Conclusions

HTHQ exhibits protective effects against l-DOPA-induced cell death via modulation of the ERK1/2-JNK1/2-Bad-Bax system in PC12 cells. These results indicate that HTHQ may show ameliorative effects against oxidative stress-induced dopaminergic neuronal cell death, but the effect of HTHQ needs to be further investigated in an animal model of PD.
